# A mini-pig model for evaluating the efficacy of autologous platelet patches on induced acute full thickness wound healing

**DOI:** 10.1186/s12917-019-1932-7

**Published:** 2019-06-07

**Authors:** Hsin-Chung Tsai, Gary Ro-Lin Chang, Hueng-Chuen Fan, Huan Ou-Yang, Li-Chuan Huang, Shinn-Chih Wu, Chuan-Mu Chen

**Affiliations:** 1 Department of Life Sciences, College of Life Sciences, National Chung Hsing University, No.250, Kuo-Kuang Road, Taichung, 402 Taiwan; 2 Department of Surgery, Taichung Hospital, Ministry of Health and Welfare, Taichung, 403 Taiwan; 3 Department of Pediatrics, and Department of Medical Research, Tung’s Taichung Metro-harbor Hospital, Wuchi, Taichung, 435 Taiwan; 4 Department of Rehabilitation, Jen-Teh Junior College of Medicine, Nursing and Management, Miaoli, 356 Taiwan; 5 Department of Animal Science and Technology, National Taiwan University, Taipei, 106 Taiwan; 6 The iEGG and Animal Biotechnology Center, and Rong-Hsing Translational Medicine Research Center, National Chung Hsing University, Taichung, 402 Taiwan

**Keywords:** Platelet concentrate, Platelet-rich plasma (PRP), Platelet-poor plasma (PPP), Platelet-rich fibrin (PRF), Platelet patch (PP), Full-thickness wound

## Abstract

**Background:**

Autologous platelet concentrates are currently widely used across different areas of regenerative medicine in order to enhance the wound healing process. Although several protocols for platelet concentrates are available, their application remains difficult due to different protocols leading to distinct products with vary potential biological uses. In this study, we attempted to make a platelet patch (PP) using mixtures of platelet rich plasma (PRP) injection and platelet rich fibrin (PRF) to promote wound repair and regeneration.

**Results:**

Experiments were performed using a full-thickness wound model in mini-pigs. Autologous PRP, PRF and PP were prepared immediately before creating four full-thickness skin wounds in pigs. We quantified concentrations of platelets, thrombin and various growth factors to ensure that the desired effect can be produced. After surgery, hydrocolloid dressing, PRP injection, PRF and PP was applied to experimentally induced wounds. Application efficacy was evaluated by measurement of wound sizes and histological examination. The results indicated that all wounds showed a significant size reduction. Wound repair efficacy in response to PP treatment exhibited enhanced re-epithelialization compared to PRP and PRF (*P* < 0.05) and higher wound contraction than did PRF application (*P* < 0.05). Another aspect, experiment using DsRed transgenic pigs as blood donors demonstrated that leucocytes in PP were incorporated into the wound bed at the end of the study, suggesting that leucocytes activity is stimulated in response to PP application. Safety of the experimental processes was also confirmed by examination of organ biopsies.

**Conclusions:**

We used a mini-pig model to evaluate the efficacy of lab-made PP on induced full-thickness wound healing. Results demonstrated that application of one piece of PP was enough to obtain comparable efficacy versus general utilization of PRP or PRF for wound care. We also demonstrated that leucocytes in PP were incorporated into the wound bed and no safety concerns have been found in the whole experiment. This study provides a novel and feasible method for veterinary or clinical wound care.

## Background

Wound healing is complex and involves an orderly and sequential series of physiological and molecular events. This process is initiated by hemostasis and followed by inflammation, cell recruitment, migration, proliferation, and tissue remodeling and maturation, which are regulated by a variety of cells, cytokines and growth factors. In chronic wounds, healing is stalled by systemic or local factors, such as diabetes, venous or arterial diseases, persistent infections, and underlying metabolic deficiencies [1]. Regardless of wound type, achieving efficient and optimal wound healing remains a significant challenge for wound care professionals in search of improved strategies and dressing materials.

Platelet-rich plasma (PRP), a blood-derived fraction consisting of platelets and growth factors in high concentrations, is gaining attention in different fields for facilitating muscle recovery from sport injuries and accelerating wound healing in acute and chronic ulcers, musculoskeletal tissue regeneration and so on [2–5]. General preparation of autologous PRP adopts one or two centrifugations, by which the first centrifugation results in three layers that include red blood cells at the bottom, an intermediate layer of buffy coat consisting of highly concentrated platelets and leukocytes (PRP), and a supernatant containing platelet-poor plasma (PPP). The upper two layers are removed prior to a second centrifugation to obtain a highly concentrated layer of platelets (PRP) after most of the PPP layer is removed. Thrombin and calcium are then added to activate fibrinogen polymerization, resulting in the concentrated platelet gel embedded in a fibrin network being formed and ready for use [6, 7]. Current standards for autologous PRP use and clinical efficacy refer to its concentration of platelet (> 10^6^ platelets/μL) and a 3- to 5-fold increase in the concentrations of growth factors [8, 9]. To date, numerous point-of-care preparation protocols and devices that have been developed are commercially available. Platelet-rich fibrin (PRF) is the second generation of platelet concentrate developed by Dohan et al. [7] Compared to PRP, PRF is prepared in a single centrifugation without the use of anticoagulants, and the thrombin in whole blood triggers the activation of platelet and fibrinogen polymerization during centrifugation to form a fibrin clot. In this clot, platelets, along with cytokines, growth factors, and leukocytes are concentrated to yield maximal efficacy in healing and tissue regeneration [10, 11]. The stronger mechanical strength of PRF protects growth factors from proteolysis [12–15], supporting extended release [11]. PRF has been utilized in a variety of fields to enhance healing of soft tissues, chronic leg ulcers and to promote bone regeneration with positive outcomes [16–19].

The considerable interest in utilizing PRP and PRF for wound care are supported by the rationale that platelets are a reservoir of various growth factors critical for tissue repair and regeneration and possess antibacterial properties in traumatic injuries [20, 21]. Therefore, the aim of this study was to combine PRP and PRF to form a novel material, i.e., platelet patch (PP), and compare its efficacy in acute wound healing to traditional PRP and PRF. Wound healing using these platelet-derived products was also compared to the current treatment using commercialized hydrocolloid dressings. Creation of full thickness cutaneous wounds in animals is an ideal model to study the healing effects of PRP. Increasing studies have been reported in dog [22–24], goat [25], horse [26, 27], and other animals like rabbit [28] and rodent species [29, 30]. In this study, we created full thickness wounds in mini-pigs as a model for acute wound healing. The similarities between pig and human skin make the mini-pig an accurate model for exploring cutaneous wound healing in humans. Concentrations of platelets and growth factors were determined before application, and objective wound size measurement and histological examination were performed to evaluate the efficacy of different treatments. Leucocytes in PP were examined by immunofluorescence staining and immunohistochemistry to determine if they were viable in the wound bed at the end of study. We also conducted a safety validation in this study.

## Results

### Concentration of platelets, thrombin and growth factors in PRP and PRF membranes

**Fig. 1:**
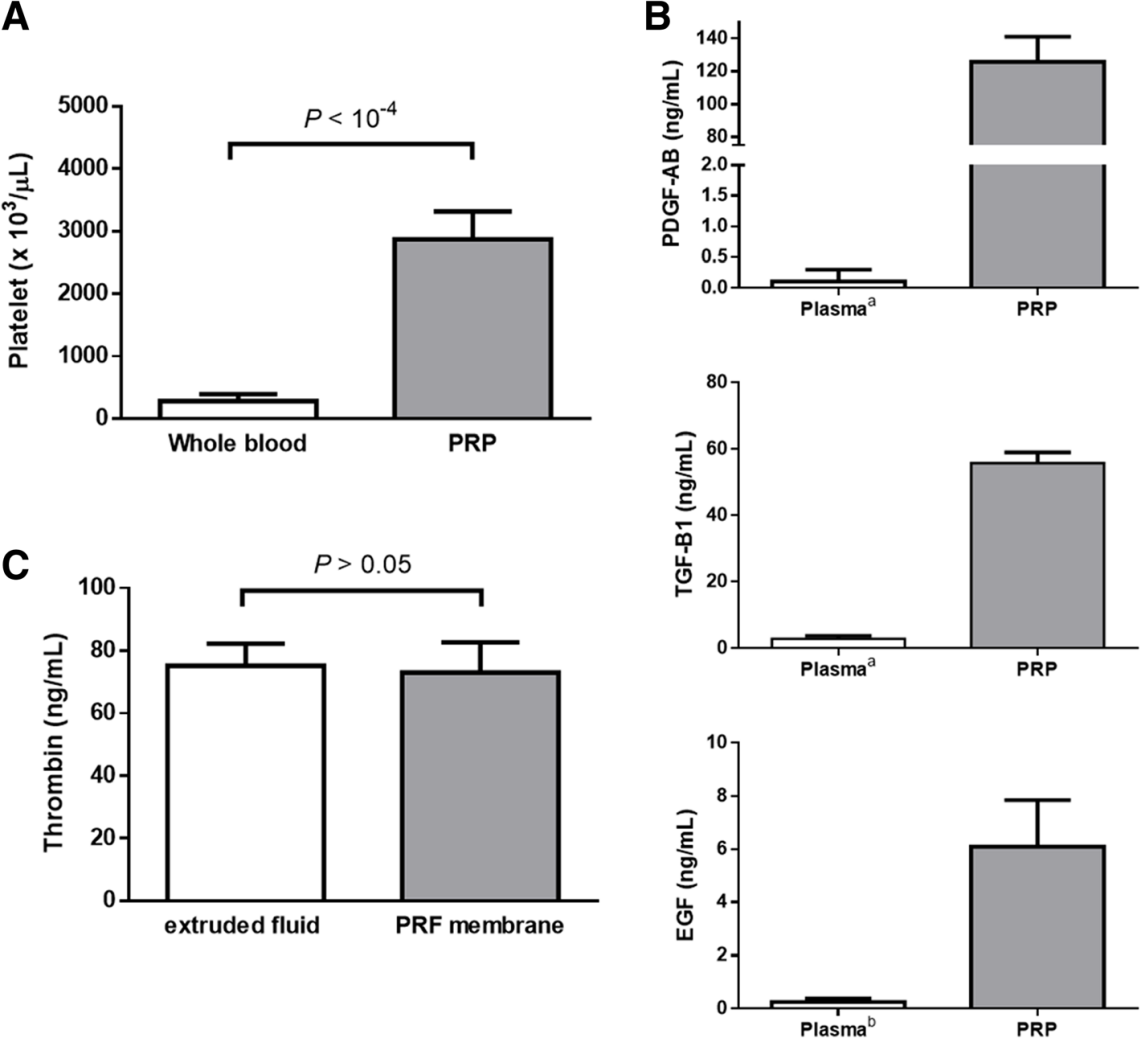
Quantification of platelets, thrombin and growth factors. **a** Platelet concentrations in PRP were significantly increased compared to whole blood (*P* < 10^− 4^). **b** The growth factors PDGF-AB, TGF-β1 and EGF in present PRP were quantified using commercialized ELISA kits. Concentrations of PDGF-AB and TGF-β1 were compared to reported baseline levels in the study of Betsch et al. (superscript a), [35] and the concentration of EGF was compared to the reported baseline levels in the study of Gomez-Caro et al. (superscript b). [36] **c** Based on protocol 1, the fibrin clot was pressed to form a PRF membrane. The concentrations of thrombin in the PRF membrane and exudate were quantified using commercialized ELISA kits

The concentration of platelets and important growth factors in the activated PRP were determined in advance in order to ensure the efficacy of the application on the wounds. As shown in Fig. 1a, platelet concentrations in freshly collected whole blood varied inter-individually between 136 × 10^3^ and 412 × 10^3^ platelets /μL (mean 283 ± 112 × 10^3^) and between 2474 × 10^3^ and 3628 × 10^3^ platelets /μL (mean 2868 ± 447 × 10^3^) in the activated PRP. The platelet concentration increased significantly in the activated PRP (*P* < 0.0001), and an average 10-fold increase was obtained using the present protocol of PRP collection. Furthermore, concentrations of growth factors in the activated PRP varied from 109 to 148 ng/mL (mean 125.8 ± 15.3) for PDGF-AB, 51–60 ng/mL (mean 55.8 ± 3.3) for TGF-β1, and 2.8–7.5 ng/mL (mean 6.1 ± 1.8) for EGF (Fig. 1b).

According to protocol 1, a fibrin clot formed after centrifugation. The fibrin clot was then pressed to produce PRF membrane and exudate. Thrombin concentrations ranged from 62 to 88 ng/mL (mean 73.0 ± 9.7) in PRF membrane, and 65 to 84 ng/mL (mean 75.2 ± 7.1) in the exudate (Fig. 1c).

### Assessment of wound healing on autologous platelet concentrates

**Fig. 2:**
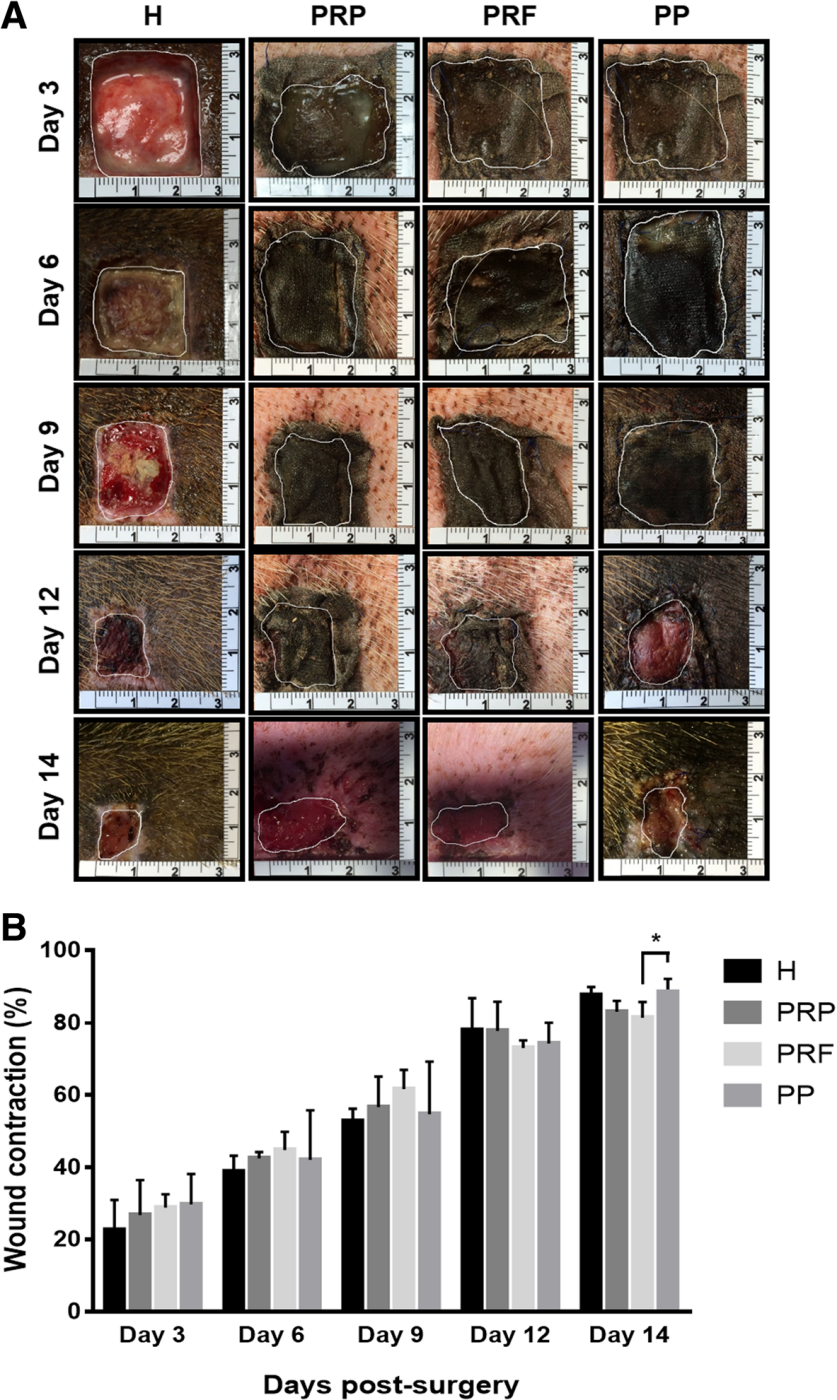
Assessment of wound closure. Wounds were imaged on days 3, 6, 9, 12 and 14. Actual wound sizes were calculated using ImageJ software. **a** Wound areas were marked by a white line in all representative images. **b** Percentages of wound contraction are expressed as the mean ± standard deviation from three independent animal experiments. Statistical analysis was performed using Student’s two-tailed *t*-test, *P < 0.05

All wounds were similar in size (9 cm^2^) at the beginning, and wound sizes were measured at intervals of 3 days until the end of the study (day 14). The results in Fig. 2a demonstrate that wound sizes gradually reduced in each group, with no significant differences found between the groups. Actual measurement of the wound sizes is shown in Fig. 2b, demonstrating significant wound size reduction in all groups. On day 14, the averaged wound sizes were measured as 1.11 ± 0.17 cm^2^ in H group for commercial hydrocolloid dressing, 1.52 ± 0.22 cm^2^ in PRP group, 1.67 ± 0.32 cm^2^ in PRF group and 1.17 ± 0.15 cm^2^ in PP group. Compared to day 0 (9 cm^2^), the wound contraction percentages at the end of the study were 87.7 ± 1.9% in H group, 83.1 ± 2.5% in PRP group, 81.4 ± 3.6% in PRF group and 87.0 ± 3.0% in PP group (Fig. 2b).

### Histological evaluation of wound healing quality

**Fig. 3:**
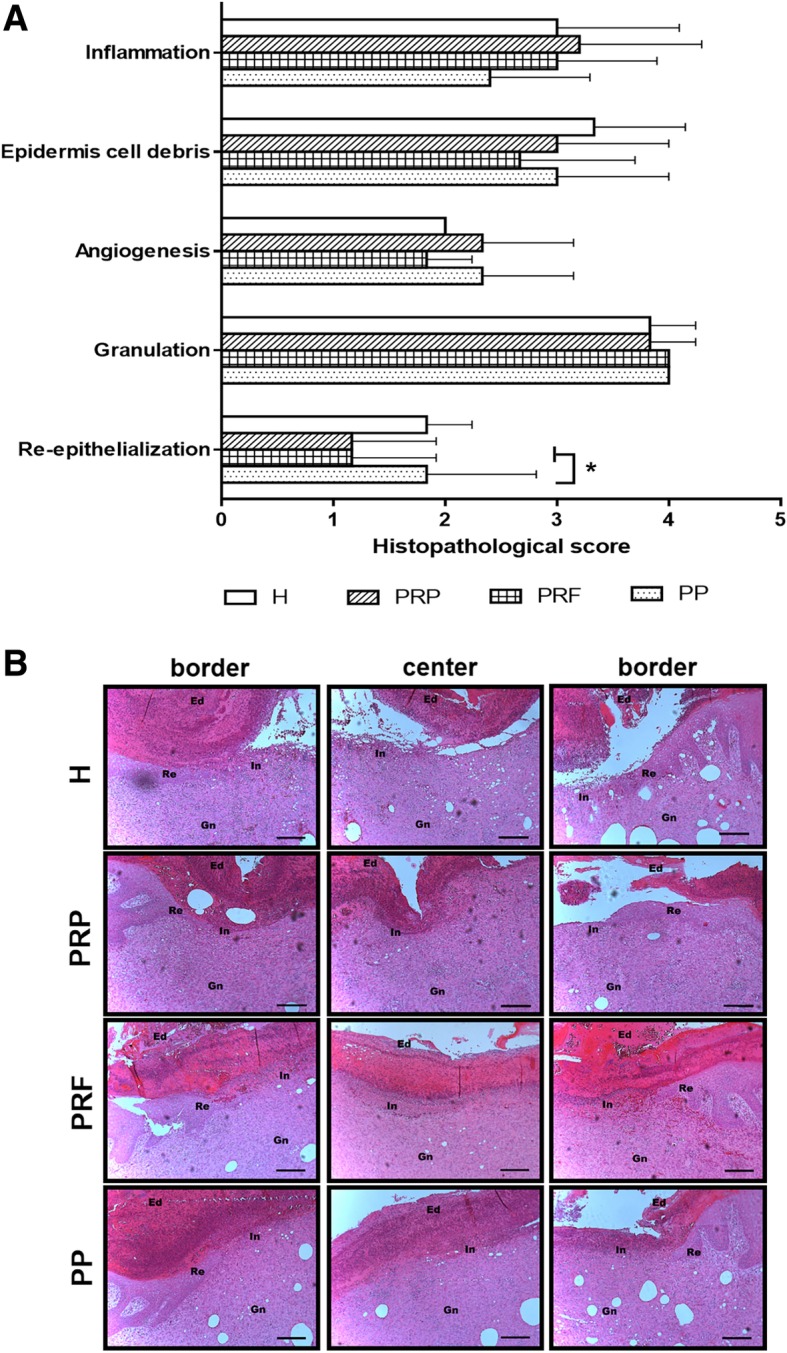
Histopathological evaluation of wound healing. **a** Histological assessment of wounds. The histological parameters of inflammation, epidermis cell debris, angiogenesis, granulation and re-epithelialization are scored from 1 to 5 according to previous report by Altavilla et al. [46] Values are presented as the mean ± standard deviation. Statistical analysis was performed using Student’s two-tailed *t*-test. There was a significant difference in re-epithelialization scores between the PP (*P* < 0.05) and PRF and PRP groups. **b** Representative H&E staining images of wound tissue sections. Ed, epidermal cell debris; In, inflammation; Gn, granulation tissues; Re, re-epithelialization. Scale bar, 200 μm

On day 14, histological evaluation of wound healing according to the degree of inflammation, epidermal cell debris, angiogenesis, granulation tissues, and re-epithelialization were performed (Fig. 3). The degree of inflammation and epidermal cell debris in all groups scored from 2 to 4, showing slight to moderate/marked with no significant differences, although inflammation in the PP group appeared to be reduced compared to other groups. Formation of granulation tissue is a major event in the proliferation phase, occurring approximately 4 days after wounding [31]. Abundant granulation tissues scoring from 3 to 4 were observable in all skin sections across each treatment at the end of study (Fig. 3b). Angiogenesis is another important event that occurs in the proliferation phase, which is necessary to sustain the newly formed granulation tissues. Most histological sections from each group showed slight (score = 2) angiogenesis. Re-epithelialization was also seen in all groups at scores of 1 to 2; however, the PP group exhibited a significantly higher score (*P* < 0.05) compared to both PRP and PRF groups (Fig. 3a).

### Survival of leucocytes in the wound beds

**Fig. 4:**
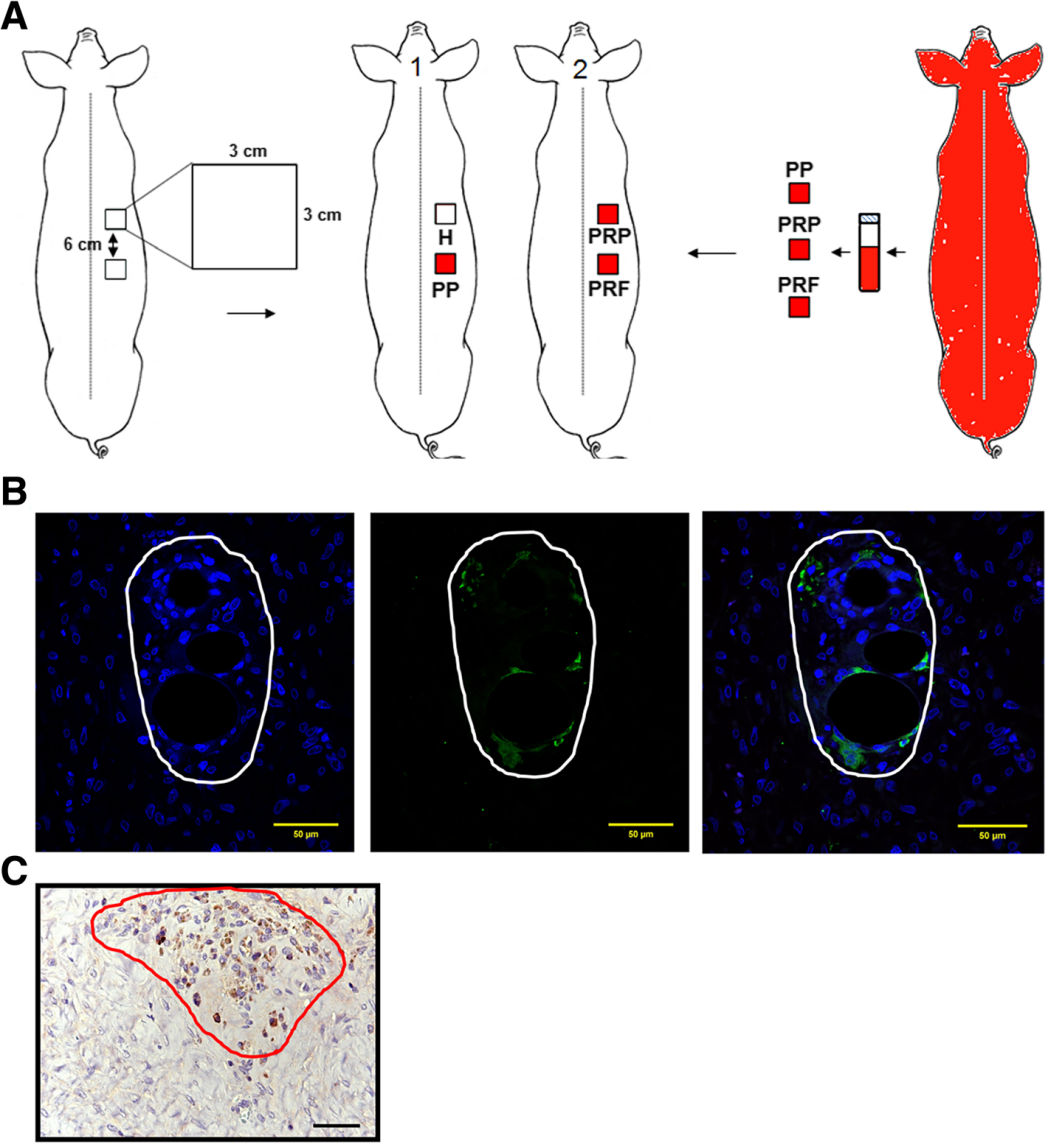
Immunostaining of donor leucocytes of PP group in the wound bed. **a** Preparation of PP, PRP, and PRF from DsRed-transgenic mini-pigs. **b** Immunofluorescence staining. Donor leucocytes in the PP in the wound bed were probed with anti-DsRed antibody and FITC-conjugated secondary antibody (middle). Cell nuclei were visualized with DAPI (left). The merge is shown on the right. The region of FITC-positive cells is circled with a white line. **c** Immunohistochemistry. The region of donor leucocytes circled with a red line was distinguished using anti-DsRed antibody and peroxidase-conjugated secondary antibody. Scale bar, 50 μm

The PP concentrate obtained in our protocol is composed of a high quantity of platelets and leucocytes. In the experiments using DsRed-transgenic mini-pigs as blood donor (Fig. 4a), some cells in the granulation tissue of the wound bed were recognized by anti-DsRed antibody, which are shown in green by coupling to FITC-conjugated secondary antibody (Fig. 4b). IHC results were also consistent with immunofluorescence by staining DsRed-containing cells in brown (Fig. 4c). These results demonstrate that the leucocytes contained in the prepared platelet concentrate survive for at least 14 days after application.

### Safety validation

**Fig. 5:**
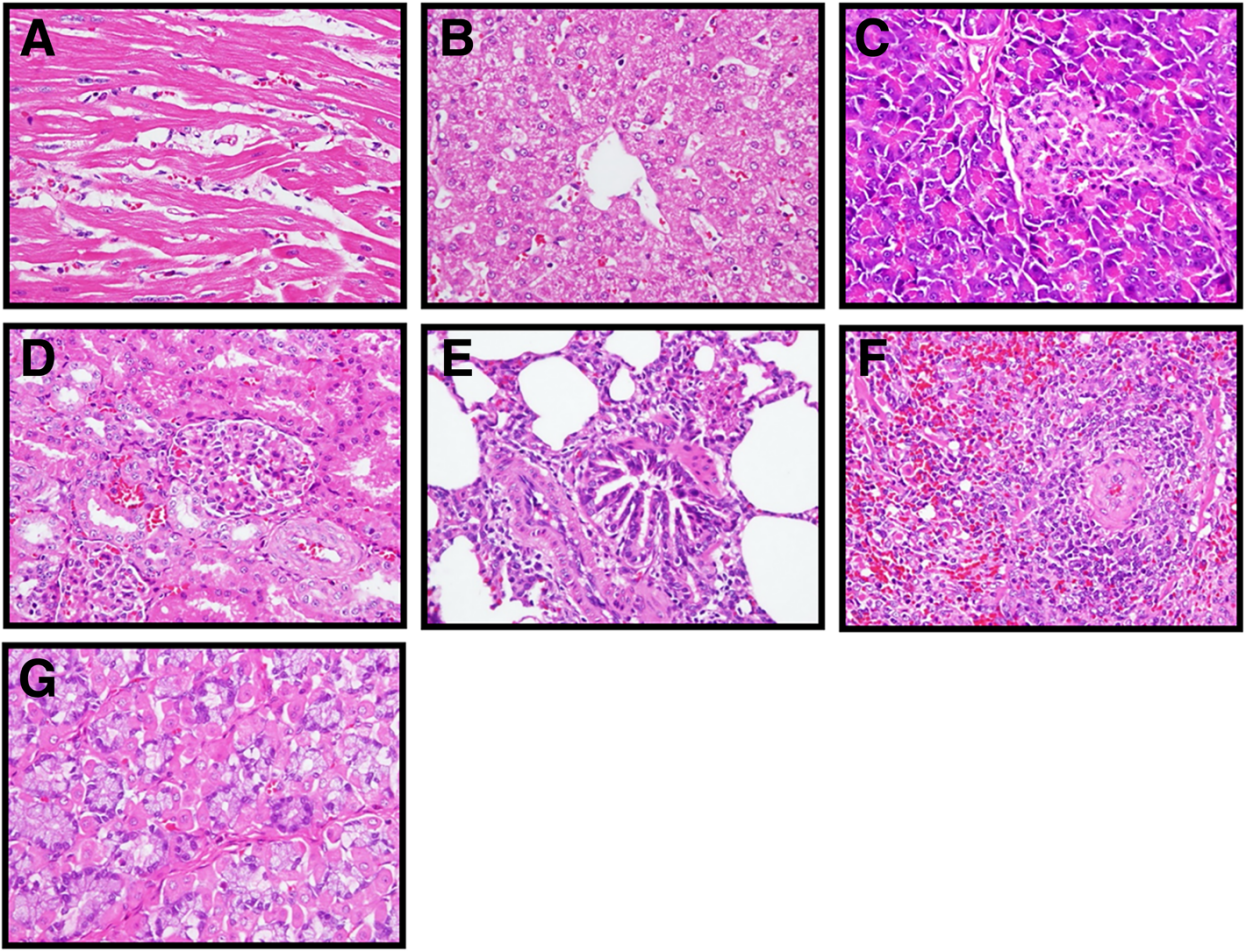
Safety validation. A series of organ biopsies, including **a** heart, **b** liver, **c** pancreas, **d** kidney, **e** lung, **f** spleen, and **g** stomach, were submitted to H&E staining for safety validation. Magnification, 400X

**Fig. 6:**
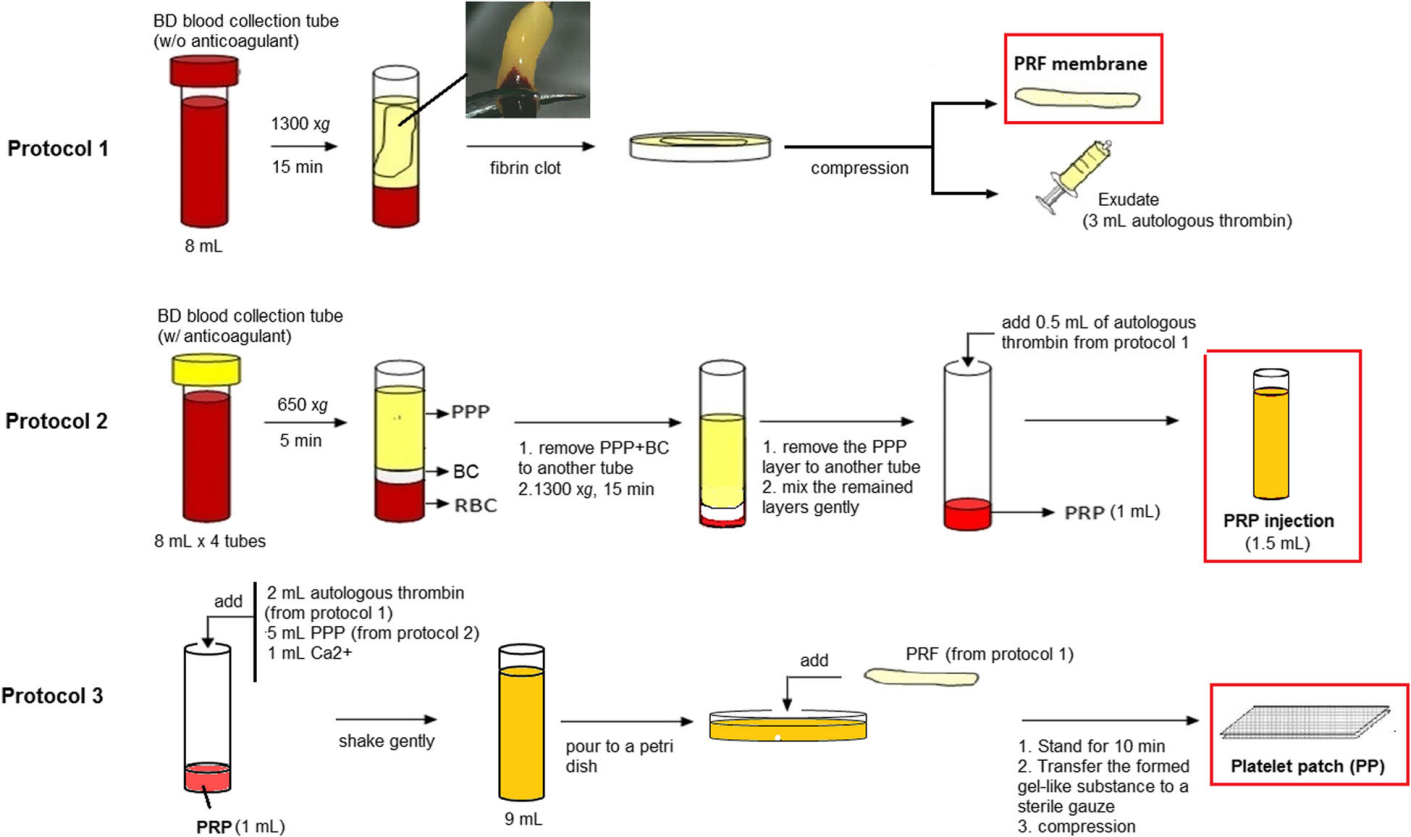
A schematic description of point-of-care preparation of PRF, PRP injection, and platelet patch (PP)

After the end of the study, a pair of mini-pigs were sacrificed for safety validation. Present results demonstrated that no pathological characteristics were found in the sacrificed mini-pig’s organ sections, including heart, kidney, liver, lung, pancreas, spleen and stomach (Fig. 5), suggesting that our lab-made PRP, PRF and PP products and the preparative methods shown in Fig. 6 are safe for clinical application.

## Discussion

Since the first report in 1997 by Whitman et al. using autologous platelet gels to replace fibrin glue in oral and maxillofacial surgery [32], the use of platelet concentrates to improve healing has been considerably investigated. According to leucocyte and fibrin content, Dohan et al. classified platelet concentrates into four categories, i.e., leucocyte-poor or pure platelet-rich plasma (P-PRP), leucocyte- and platelet-rich plasma (L-PRP), leucocyte-poor or pure platelet-rich fibrin (P-PRF) concentrates and leucocyte- and platelet-rich fibrin (L-PRF) concentrates [33]. Several automated and manual protocols for these platelet concentrates are currently available [34]; however, most of them are either cost-ineffective or time-consuming. In this study, we use a method analogous to that of commercialized Curasan’s PRP kit for point-of-care preparation of L-PRP (Fig. 6, protocol 2) [35]. After a two-step centrifugation procedure, most of the PPP layer is discarded, and the final PRP concentrate is composed of a high quantity of platelets and leucocytes, as well as residual RBCs. This concentrate can be applied after activation with thrombin from an autologous or heterologous origin. Here, we also compared L-PRP with another type of platelet concentrate, i.e., L-PRF, which is modified from Choukroun’s PRF [6], a second-generation platelet concentrate. For point-of-care preparation of L-PRF, blood is collected without anticoagulants, and only one-step centrifugation is needed (Fig. 6, protocol 1). Platelet activation and fibrin polymerization are triggered during centrifugation, and a PRF clot in the middle layer is formed after centrifugation. The PRF clot can be pressed to become a strong membrane for application. This protocol is the simplest and least expensive developed so far. Furthermore, we combine the use of PRP and PRF to determine whether an additive effect occurs (Fig. 6, protocol 3). From a technical view, all of the present protocols can be performed within half an hour, and expensive centrifuge and preparation kits are not required. Hence, their use in daily practice might be feasible.

The working definition of PRP is that the platelet concentration must be more than 10^6^/μL in 5 mL of plasma or five times higher than normal baseline. Lower concentrations may not improve the wound healing, and greater concentrations do not necessarily have an improved effect [8]. Platelet counting confirmed that the present protocol for PRP preparation is indeed able to meet this standard to have a therapeutic effect (Fig. 6). Before application, the PRP needs to be activated to create a platelet-fibrin matrix. Several commercial preparations of PRP use bovine thrombin as an activator; however, it may cause development of antibodies to some clotting factors and thrombin, increasing unwanted coagulation problems. In this study, we use autologous thrombin to activate PRP. The autologous thrombin originated from the extruded fluid of PRF fibrin clot in present protocol 1, where the extruded fluid was confirmed to have the content of thrombin nearly equal to the pressed PRF membrane (Fig. 1c). Under normal conditions, the concentration of thrombin in the blood is very low to avoid abnormal coagulation; therefore, our results confirm that platelet activation occurs naturally during centrifugation and does not require the addition of an anticoagulant to increase the concentration of thrombin. Platelets are the most important regulators of wound healing, not only because they release many clotting factors, but also because they release a number of potent growth factors such as PDGF, TGF-β1, and EGF. Here, we quantified PDGF-AB, TGF-β1 and EGF concentrations after thrombin activation and found that these growth factors in our PRP were more than ten times higher than porcine baseline levels shown in the studies of Betsch et al. [36] (PDGF, 107.7 ± 194.5 pg/mL and TGF-β1, 2899.7 ± 855.5 pg/mL) and Gomez-Caro et al. [37] (EGF, 0.25 ng/mL) (Fig. 1b). These growth factors are released from α-granules of platelets and play central roles during wound healing processes. For example, PDGF is a potent attractive agent of macrophages and neutrophils that promotes tissue repair, cell proliferation, matrix formation and remodeling. TGF-β activates monocytes into macrophages, increasing inflammation and tissue debridement and promotes matrix formation and remodeling. EGF promotes fibroblast migration and proliferation, resulting in re-epithelialization and angiogenesis [34]. We did not quantify other important growth factors, specifically vascular endothelial growth factor (VEGF) or fibroblast growth factor (FGF), but both of them can induce angiogenesis and collagen synthesis as well [34].

As mentioned above, present platelet concentrates belong to leucocyte-rich PRP and leucocyte-rich PRF. The role of leucocytes in these platelet concentrates and their contribution to the observed overall effect remains unclear. Some studies have noted their antimicrobial effects and immune regulation [38]. Apart from anti-infection, Werther et al. reported that leucocytes produce large amounts of VEGF [39]. In this study, we found the leucocytes in PP were incorporated into the wound bed. Moojen et al. reported that the strong antimicrobial effect of their platelet leucocyte gel (a kind of L-PRP) was limited to the first hour after application [38]. Therefore, the long life of autologous leucocytes in the wound bed may help the wound apart from bacterial contamination, providing long-term protection and releasing more VEGF-inducing angiogenesis. However, these hypotheses need to be further tested.

In clinical and animal studies, both PRP and PRF have been proven individually to yield a positive effect on wound healing. In this study, we tested whether PRP and PRF cause different effects on wound healing and if a better effect could be obtained after combining PRP and PRF, and we also compared their healing effects with a hydrocolloid dressing used in clinic practice. According to the actual measurement of wound sizes, all treatments produced similar results during the healing process, and each group achieved > 80% wound contraction by the end of the study (Fig. 2). The use of autologous PP was similar to the treatment using commercial hydrocolloid dressings (treatment H) and showed better results compared to PRP and PRF at the end of the study (day 14), but significant difference (*P* < 0.05) was only seen versus PRF. Histological biopsy examination also confirmed that there was no significant difference in most of the scores for histological parameters between the treatments (Fig. 3). Notably, treatment with PP showed a higher score of re-epithelialization than that with PRF and PRP (*P* < 0.05) and was comparable with the use of hydrocolloid dressing. Nevertheless, there is one thing that deserves our attention. The limited filling of the wound cavity with one piece of platelet patch (PP) was not sufficient to induce formation of abundant granulation tissue. Therefore, using more platelet patches to cover the wound cavity may promote faster growth of the granulation tissues of the acute wounds and provide an alternative to clinical wound management. If post-operative care were supported by splinting, negative pressure wound therapy (NPWT) or hyperbaric oxygen (HBO), results should be more conspicuous.

Examination of organ histology confirmed the safety of this experiment process, supporting its future application in patients. We demonstrated that this lab-made method yields good biomaterials such as scaffold (platelet patch, PRF), cells and signals (GFs), which are the golden triangle of tissue engineering. In our ongoing and future experiments, we want to know whether platelet patches provide the best skin grafting during different post-operative periods. In conjunction with other stromal cells such as adipose-derived stem cells (ADSC), we hope to provide a more powerful tool to treat difficult wound healing in the clinical environment.

## Conclusion

In this study, we successfully established a full-thickness wound model in mini-pigs to evaluate a lab-made platelet patch (PP) using mixtures of PRP injection and PRF fibrin to promote wound repair and regeneration. Wound repair efficacy in response to PP treatment exhibited enhanced re-epithelialization and higher wound contraction. Using DsRed transgenic pigs as blood donors, we further demonstrated that leucocytes in PP were incorporated into the wound bed. Safety of the experimental processes was confirmed by examination of organ biopsies. Only one piece of PP was applied to cover the wound cavity in this study, suggesting that improved healing may result if more pieces of PP are used. Therefore, this study provides a novel and feasible method for veterinary or clinical wound care.

## Methods

### Animals

Six mini-pigs including two ones that carried a DsRed-monomer reporter gene as described previously [40], aged approximately 4 months, were provided by National Taiwan University (NTU) (Taipei, Taiwan). Animal experiments were reviewed and approved by the Institution Animal Care and Use Committee (IACUC105089) of NTU. All pigs were housed under standard environmental conditions (23 ± 2 °C, with 55 ± 5% humidity) with free access to lab chow and water.

### Preparations of autologous PRF membrane, PRP injection and platelet patch (PP)

As shown in Fig. 6, a total of 40 mL of whole blood was drawn from the internal jugular vein of each mini-pigs and was immediately used for the preparation of autologous PRP, PRF and PP.

To prepare the PRF membrane, 8 mL of whole blood was centrifuged (collected in a serum red-capped BD Vacutainer™ tube; Becton Dickinson, Oakville, ON, USA) at 1300×*g* for 15 min in a bench-top centrifuge (CN-1040; Hsiangtai Machinery IND. Co., Taipei, Taiwan). Three layers were generated, including red corpuscles at the bottom, a structured fibrin clot in the middle, and acellular platelet poor plasma (PPP) at the top. The fibrin clot was collected and pressed between two pieces of gauze, and a ready-to-use PRF membrane was obtained. The exudate containing autologous thrombin was collected for use with a syringe (Protocol 1). This protocol was modified from Choukroun’s method of collecting PRF [41].

To prepare PRP injections, 32 mL of whole blood (collected in four yellow-capped BD Vacutainer™ tubes with 0.15 mg/mL anticoagulant citrate phosphate dextrose) was centrifuged at 650×*g* for 5 min, forming three layers. Next, both the top layer of PPP and the middle layer of buffy coat (BC) were transferred to a new sterile tube for a second centrifugation at 1300×g for 15 min. Three new layers were generated, from which most PPP at the top was removed and used elsewhere, while the remaining PRP (some PPP + BC + residual RBC, 1 mL) was activated with 0.5 mL of autologous thrombin from protocol 1 to produce the PRP injection (Protocol 2). The PRP injection (1.5 mL) was used for direct injection at the periwound area. This protocol was modified from Curasan’s method of collecting PRP [35].

In this study, we combined PRP with PRF to produce platelet patches (PP) for wound treatment. To prepare PP, the PRP (1 mL) was activated with 5 mL of PPP (collected in protocol 2), 1 mL of calcium gluconate (Glucal Black Injection, Union Chemical & Pharmaceutical Co., LTD, Taiwan) and 2 mL of autologous thrombin (from protocol 1) [42]. The mixture was shaken gently and then transferred to a Petri dish. Next, PRF membrane was added and 10 min later, the gel product was pressed between two silver dressings (Atrauman® Ag; Hartmann, Heidenheim, Germany) to form a piece of PP (protocol 3).

### Determination of platelet concentrations

Platelet concentrations in whole blood and in activated PRP were measured by Union Clinical Laboratory (http://www.ucl.com.tw, Taipei, Taiwan) using an automated hematological analyzer (XE-2100, Sysmex Corp., Japan).

### Determination of thrombin concentrations

The concentration of thrombin in PRF membranes and in the exudates of fibrin clots were determined using an enzyme-linked immunosorbent assay (ELISA) kit (Cat. KA1407; Novus Biologicals, Littleton, CO, USA).

### Determination of growth factors concentrations

Growth factors, including platelet-derived growth factor (PDGF), transforming growth factor-β1 (TGF-β1) and epidermal growth factor (EGF) in the activated PRP, were quantified using ELISA kits Cat. MBS706643 (MyBioSource, Inc., San Diego, CA, USA), Cat. GR106126 (Genorise Scientific Inc., Glen Mills, PA, USA) and Cat. E0560P (EIAab Inc., Maryland, USA), respectively.

### Creation of full-thickness skin wounds in mini-pigs and subsequent treatments

**Fig. 7:**
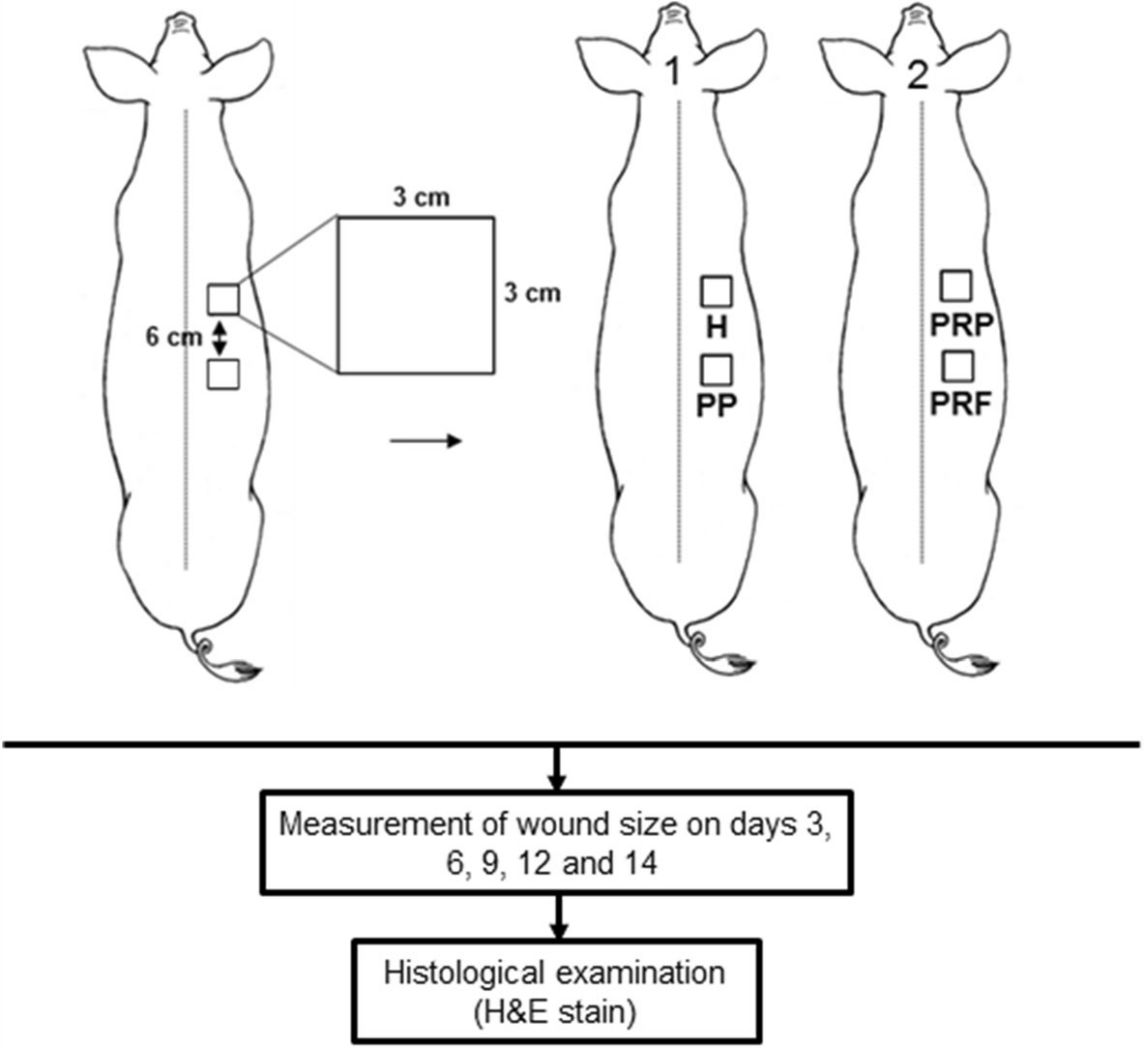
Acute full-thickness skin wound model. Six mini-pigs were used in this study, and two mini-pigs were paired for creation of two 3 × 3 cm^2^ full-thickness wound defects (6 cm apart from each other) on the right dorsolateral area of the trunk of each mini-pig. As indicated, hydrocolloid dressing (H), PRP injection (PRP), PRF, or platelet patch (PP), was then applied to the wounds. After application, wound sizes were recorded on days 3, 6, 9, 12 and 14. Subsequently, histological examination was performed at the end of the study. Animal experiments were conducted in triplicate for this study

After 1-month acclimation, six mini-pigs with mean body weights of 26.6 ± 4.1 kg, were subjected to surgery to create full-thickness wounds. Prior to surgery, mini-pigs were anesthetized by ketamine/xylazine (KX) sedation and followed by inhalation anesthesia, and 40 mL of whole blood was drawn for point-of-care preparation of each kind of platelet concentrate as described in Fig. 6. The mini-pigs’ dorsal hair was removed with clippers and skin were cleaned with chlorhexidine (Merck, Darmstadt, Germany). As shown in Fig. 7, on the right dorsolateral area of the trunk with 3 cm from the spine, two squares of 3 × 3 cm^2^ separated by 6 cm were demarcated with sterile ink, and then, the skins at the marked sites were excised precisely using a No. 15 surgical blade (Zymeck, Gujarat, India) to create two full-thickness wounds with an average depth of 1.5 ± 0.2 cm without damaging the underlying muscular layer [43]. After surgery, pigs were divided into three pairs. In each pair, the first pig received application of Comfeel® hydrocolloid film (Medline Industries, Inc., Northfield, IL, USA) in the wound at the upper position (treatment H) and one piece of PP in wound at the lower position (treatment PP), the second pig received 1.5 mL PRP injection in the wound at the upper position (treatment PRP) and the application of PRF membrane in the wound at the lower position (treatment PRF). Attentively, PRP injection was injected at 6–8 different points in the periwound area with 0.5 cm from the edge of the wound, while PP, PRF membrane and commercial hydrocolloid film were placed directly on the wounds, and the bloods used to prepare PRP injection, PRF membrane and PP were all made from the same pigs that received the treatments. Animal welfare-related assessments and interventions were carried out during the experiment, including morphology, physiology, behavior, dietary intake, water consumption, and general state of health. Subsequently, all wounds were further covered with film dressing (Atrauman® Ag; Hartmann, Heidenheim, Germany) and foam dressing (Mepilex®; Mölnlycke Health Care, Norcross, GA, USA) to avoid dehydration and microbial contamination of the wound beds. Finally, each pig was given 0.03 mL per kilogram body weight of Penisol (300,000 IU/mL Procaine Penicillin G; China Chemical & Pharmaceutical Co., Taiwan) once daily for 3 days. The same experiments have been performed twice with an interval > 3 months (*n* = 3 × 2).

### Assessment of wound healing

The process of wound healing was recorded using a digital camera on days 3, 6, 9, 12 and 14 (end of study). Wounds were measured from photographs taken perpendicularly to the wound surface and included a plastic ruler in the same plane of the wounds as a comparison of their actual sizes. Wound sizes at day x (A_day x_, cm^2^) were calculated by circling the margin of the wound using free ImageJ software (https://imagej.nih.gov/ij/), and wound healing was assessed by calculating the percentage of wound contraction according to the following equation [23]:

$$ \mathrm{Wound}\ \mathrm{contraction}\ \mathrm{at}\ \mathrm{day}\ \mathrm{X}\left(\%\right)=\left[1-{\mathrm{A}}_{\mathrm{day}\mathrm{x}}/{\mathrm{A}}_{\mathrm{day}\ 0}\right]\times 100\%. $$

### Histological examination

At the end of the study (day 14), wound biopsies, including surrounding non-wounded skin, were excised, fixed and embedded in paraffin. Tissue sections were stained with hematoxylin and eosin (H & E) reagents [44, 45]. The parameters of histological evaluation included evaluating for the presence of inflammation, epidermal cell debris, angiogenesis, granulation tissue and re-epithelialization. Inflammation and epidermal cell debris was scored from 1 to 5 according to the degree of lesion as follows: 1, minimal (< 1%); 2, slight (1–25%); 3, moderate (26–50%); 4, moderate/marked (51–75%); and 5, marked (76–100%) [46]. Angiogenesis, granulation tissue and re-epithelialization was graded from 1 to 4 by the criteria adopted from Altavilla et al. [47]. All evaluation was performed by a certified veterinary pathologist in Research Center for Animal Medicine, National Chung Hsing University, Taichung, Taiwan. The scores for each parameter were averaged from six independent experiments. All evaluation was performed by a certified veterinary pathologist in Research Center for Animal Medicine, National Chung Hsing University, Taichung, Taiwan. The scores for each parameter were averaged from six independent experiments.

### Immunofluorescence and immunohistochemistry analyses

During the preparation of PRP and PRF, high quantities of platelets and leucocytes are in the buffy coat or embedded in the fibrin clot after centrifugation. To determine whether these leucocytes remained in the wound beds until the end of study, we used DsRed-transgenic mini-pigs as blood donors [40], and then, the PRP, PRF and PP prepared from them were applied to the full-thickness wounds created on non-transgenic littermate recipients with the same blood type. Blood types were confirmed in advance by mixing donor and recipient blood, and in the absence of agglutination, blood types were concluded to be the same. Except for the source of blood, the other procedures are the same as described in the above methods. Skin biopsies of wound beds were obtained on day 14 post-wounding. After adequate fixation and paraffin embedding, histological slices were cut and subjected to immunofluorescence (IF) [48] and immunohistochemistry (IHC) [49, 50] staining using rabbit anti-DsRed primary antibody (ab62341; Abcam, Eugene, CA, USA) and FITC-conjugated and peroxidase-conjugated secondary anti-rabbit antibodies (ab97063; Abcam, Eugene, CA, USA), respectively.

### Safety validation

Upon the end of the experiments, a pair of mini-pigs were randomly selected and humanely sacrificed through intravenous injection of overdose of sodium pentobarbital (100 mg/kg; Sigma-Aldrich, St. Louis, MO, USA) for autopsy to determine the safety of the present experimental procedures. Various organs, including heart, kidney, liver, lung, pancreas, spleen and stomach were examined for pathological findings in accordance with the Guidelines for Nonclinical Safety Studies of Biopharmaceuticals (Ministry of Health and Welfare, Taiwan). The remaining mini-pigs were returned to the original laboratory for another experiments.

### Statistical analysis

All values are presented as the mean ± standard deviation (SD). Statistical analysis was performed by the Student’s two-tailed *t*-test, and the difference between two groups with *P* < 0.05 was considered significant. No statistical power calculation was conducted prior to the study. The sample size was based on our previous experience with this design.

## Data Availability

All the data are present in the text.

## Abbreviations

ADSC: Adipocyte-derived stem cells
BC: Buffy coat
EGF: Epidermal growth factor
ELISA: Enzyme-linked immunosorbent assay
FGF: Fibroblast growth factor
H&E: Hematoxylin and eosin
HBO: Hyperbaric oxygen
IF: Immunofluorescence
IHC: Immunohistochemistry
L-PRF: Leucocyte- and platelet-rich fibrin
L-PRP: Leucocyte- and platelet-rich plasma
NPWT: Negative pressure wound therapy
PDGF: Platelet-derived growth factor
PP: Platelet patch
PPP: Platelet-poor plasma
P-PRF: Pure platelet-rich fibrin
P-PRP: Pure platelet-rich plasma
PRF: Platelet-rich fibrin
PRP: Platelet-rich plasma
TGF-β1: Transforming growth factor-β1
VEGF: Vascular endothelial growth factor
